# Vascular phenotype in angiogenic and non-angiogenic lung non-small cell carcinomas

**DOI:** 10.1038/sj.bjc.6600015

**Published:** 2002-01-21

**Authors:** E Passalidou, M Trivella, N Singh, M Ferguson, J Hu, A Cesario, P Granone, A G Nicholson, P Goldstraw, C Ratcliffe, M Tetlow, I Leigh, A L Harris, K C Gatter, F Pezzella

**Affiliations:** 3rd Department of Respiratory Medicine, Sismanogleiou Hospital, Sismanogleiou 1, PC 15126 Athens, Greece; ICRF Medical Statistics Group, Centre for Statistics in Medicine, Institute of Health Sciences, Old Road, Headington, Oxford OX3 7LF, UK; Department of Histopathology, University College London, Rockefeller Building, University Street, London WC1E 6JJ, UK; Division of Thoracic Surgery, Universita' Cattolica del Sacro Cuore, Largo A. Gemelli 8, 00168 Roma, Italy; Department of Histopathology, Royal Brompton, Hospital, Sydney Street, London SW3 6NP, UK; Department of Thoracic Surgery, Royal Brompton, Hospital, Sydney Street, London SW3 6NP, UK; Centre for Cutaneous Research, Barts and the London, Queen Mary's School of Medicine and Dentistry, 2 Newark St, London E1 2AT, UK; ICRF Medical Oncology Unit, Churchill Hospital, Old Road, Headington, Oxford OX3 9LJ, UK; ICRF Tumour Pathology Group, Nuffield Department of Clinical Laboratory Sciences, John Radcliffe Hospital, Headington, Oxford OX3 9DU, UK

**Keywords:** lung cancer, angiogenesis, vascular phenotype

## Abstract

We have previously described a group of non-small cell lung carcinomas without morphological evidence of neo-angiogenesis. In these tumours neoplastic cells fill up the alveoli and the only vessels present appear to belong to the trapped alveolar septa. In the present study we have characterised the phenotype of the vessels present in these non-angiogenic tumours, in normal lung and in angiogenic non-small cell lung carcinomas. The vessels, identified by the expression of CD31, were scored as mature when expressing the epitope LH39 in the basal membrane and as newly formed when expressing αVβ3 on the endothelial cells and/or lacking LH39 expression. In the nine putative non-angiogenic cases examined, the vascular phenotype of all the vessels was the same as that of alveolar vessels in normal lung: LH39 positive and αVβ3 variable or negative. Instead in 104 angiogenic tumours examined, only a minority of vessels (mean 13.1%; range 0–60%) expressed LH39, while αVβ3 (in 45 cases) was strongly expressed on many vessels (mean 55.5%; range 5–90%). We conclude that in putative non-angiogenic tumours the vascular phenotype is that of normal vessels and there is no neo-angiogenesis. This type of cancer may be resistant to some anti-angiogenic therapy and different strategies need to be developed.

*British Journal of Cancer* (2002) **86**, 244–249. DOI: 10.1038/sj/bjc/6600015 www.bjcancer.com

© 2002 The Cancer Research Campaign

## 

The nature and the role played by vessels in tumour growth have been debated for the last 50 years. The original view that tumours rely on pre-existing vessels has been overturned by robust experimental work showing that the formation of new vessels is crucial to tumour growth (Folkman, 1971, 1995). In the words of Folkman (1990): ‘Once a tumour ‘take’ has occurred, every increase in tumour cell population must be preceded by an increase in new capillaries converging on the tumour’.

However, there is now growing evidence that, in certain situations, tumours can also exploit pre-existing vessels. It has been reported by the writers and others (Kolin and Koutoulakis, 1988; Kolin, 1995; Pezzella *et al*, 1996, 1997, 2000; Offersen *et al*, 2001) that both lung primaries and secondaries can be divided into two groups, one of which shows no histological evidence of neo-angiogenesis. A pattern of non-angiogenic growth has also been described in glioblastoma multiforme (Wesseling *et al*, 1994). Hyjek *et al* (1999) have also reported a remarkably low number of immature vessels in indolent lymphomas compared to the increased number of newly formed vessels in high grade lymphomas.

We have reported that lung carcinomas without angiogenesis are characterized by lack of parenchymal destruction and absence of new vessels and tumour associated stroma. The only vessels present appear to be those in the alveolar septa and their presence highlights, throughout the whole tumour, the lung alveoli filled up by the neoplastic cells. In a first study, clinico-pathological correlation suggested that these tumours are highly aggressive (Pastorino *et al*, 1997) though a subsequent study (Offersen *et al*, 2001) suggests that this is not the case and that these tumours are probably less aggressive than initially thought. However, in both papers the confidence intervals of the relative risk for patients with non-angiogenic tumours are very wide leaving the problem of the prognosis of these tumours open. Because of their ability to exploit pre existing vessels and the lack of neo-angiogenesis, these tumours are likely to have some major biological differences from angiogenic neoplasms. However, our observation was based on morphological observations and only suggests, but does not demonstrate, that some tumours could grow without the presence of newly formed vessels.

Three recent studies (Nagano *et al*, 1993; Holash *et al*, 1999a,b) further support the hypothesis that some tumours could grow by exploiting pre-existing vessels. Vessel cooption has also been described in uveal melanoma (Maniotis *et al*, 1999). In the same study the authors propose that in this type of melanoma also a different type of angiogenesis-independent neoplastic growth named ‘vasculogenic mimicry’, occurs. In vasculogenic mimicry; the same melanoma cells would form ‘channels’ within the tumour compartment.

Further investigation is required to establish whether there is a type of tumour truly able to exploit pre-exiting vessels and grow in the absence of angiogenesis. There are several possible approaches to resolve this issue such as the use of ‘*in vitro*’ angiogenic assays and *in-situ* detection of angiogenic factors. In this study we have chosen to investigate the basic phenotype characteristics of both the intra-tumour and normal lung vessels. Our aim was to establish whether the vessels present in putative non-angiogenic tumours have a ‘mature’ phenotype, as in the normal lung, or an ‘immature’ phenotype as in angiogenic tumours.

To distinguish between mature and immature vessels we looked at the expression or loss of two markers. The first marker is an epitope within the lamina lucida of the basement membrane of human tissues (Almeida *et al*, 1992a) identified by the antibody LH39. This molecule is expressed on the basal membrane of capillaries and small venules in a variety of normal human tissues but it is absent in small vessels present in pyogenic granulomas or non-specific oral ulceration (Almeida *et al*, 1992a,b). Furthermore, Kakolyris *et al* (2000) have demonstrated that in normal breast tissue (an organ in which remodelling occurs physiologically) a proportion of the vessels is LH39 negative. These data suggest that the detection of LH39 allows the discrimination between mature and recently formed vessels. Most of the intra-tumoural vessels should therefore be negative for LH39 and three studies, on oral carcinomas (Almeida *et al*, 1992b), on breast carcinomas (Kakolyris *et al*, 2000) and on lung cancer (Kakolyris *et al*, 1999) have confirmed this hypothesis. Even in the case of breast cancer, the number of LH39-negative intra-tumoural vessels is significantly higher than in normal breast tissue (where some LH39 negative, immature vessels are present because of the physiological remodelling of the tissue).

The other marker we looked at, on the endothelium, is the integrin αVβ3. We looked at it mainly for two reasons.

The first one is that αVβ3 has been reported to be expressed on newly formed endothelium (Brooks *et al*, 1994) but some authors disagree (Rabb *et al*, 1996; Navratil *et al*, 1997; Pazouki *et al*, 1997): they have demonstrated its expression also on a variety of normal tissues. Therefore we wanted to establish whether in the lung αVβ3 was expressed only in intra-tumour vessels or also in normal tissue vessels. Eventually we wanted to establish whether the αVβ3 pattern of expression in putative non-angiogenic tumours was like the one present in normal lung or in lung carcinomas.

The second reason is that Friedlander *et al* (1995) have described also an αVβ3-independent angiogenic pathway. Therefore while the uniform expression, or non-expression, on intra-tumour vessels of αVβ3 could suggest the activation of just one angiogenic pathway, the mixed presence of αVβ3 positive and negative immature vessels would argue in favour of different pathways simultaneously activated.

We also attempted to investigate the expression on the endothelium of αVβ5 which is expressed in newly formed endothelium in an alternative fashion to αVβ3 (Friedlander *et al*, 1995). However, αVβ5 turned out to be too widely expressed making the evaluation of endothelial staining unreliable (data not shown).

## MATERIAL AND METHODS

### Tissue samples

Tissue samples from 113 patients were available. All patients had a diagnosis of non-Small Cell Lung Carcinoma (n-SCLC) and had undergone radical surgical resection either in Rome (Department of Thoracic Surgery, Catholic University) or in London (Department of Thoracic Surgery, Royal Brompton Hospital). Fresh tissue was snap frozen in liquid nitrogen. For all cases the diagnosis was established on routinely formalin-fixed paraffin-embedded material.

Five samples of normal lung tissue were also obtained either from lung parenchyma away from the tumour (three samples) or from patients undergoing lung resection for emphysema (two samples).

The tumours were classified as angiogenic (basal, papillary or diffuse patterns) and non-angiogenic (alveolar pattern) as previously described (Pezzella *et al*, 1997).

### Immunocytochemistry

The following antibodies were used: the anti-CD31 JC70 monoclonal antibody (Parums *et al*, 1990) staining endothelial cells, the anti lamina lucida antigen LH39 antibody (Almeida *et al*, 1992a) and the anti αVβ3 antibody LM609 (Chemicon International).

All immunostaining was performed on frozen tissue sections. For single immunostaining the primary antibody was incubated for 1 h at room temperature. Labelling was performed with an avidin-biotin peroxidase labelling system (DAKO Duet). The appropriate secondary antibody was applied for 35 min, after which the DAKO streptavidin-biotin complex was applied. Finally, application of DAB solution developed the staining reaction.

The double immunostaining, using the anti CD31 and anti LH39 antibodies, was performed as follows. Firstly the anti LH39 antibody was incubated for 1 h at room temperature. Labelling was performed with an avidin-biotin peroxidase labelling system (DAKO Duet). The appropriate secondary antibody was applied for 35 min, after which the DAKO streptavidin-biotin complex was applied. Application of DAB solution developed the staining reaction. After rinsing, the sections were incubated overnight with anti CD31. After further rinsing sections were incubated with rabbit-anti-mouse immunoglobulin and finally with APAAP complexes.

### Evaluation of tumour vascularity

To evaluate the proportion of mature vessels, 200 vessels were counted in each slide on which double immunostaining for CD31 and LH39 had been carried out. The Vascular Maturation Index (VMI), defined as the percentage of vessels with LH39 positive basal membrane (Kakolyris *et al*, 1999), was then calculated.

On a similar basis we derived an Immaturity Index in order to compare vessels positive for αVβ3 to all the CD31 positive vessels. In order to make a direct comparison, staining was undertaken on serial sections and the count for CD31 on a section was related to the count for αVβ3 in the same areas on the serial section. In this case the index indicates the per cent of the immature vessels, i.e. αVβ3 positive, of the total of CD31 positive vessels.

### Numerical evaluation of vascular phenotype in the angiogenic tumours

Because of the small numbers involved a statistical tool as such is not employed here. Descriptive analysis is used with appropriate numerical and graphical manipulation of the available data. For the tumours where double staining was achieved and a readable immunostaining for αVβ3 was obtained, the percentages of LH39 positive and αVβ3 positive were considered separately.

## RESULTS

### Histological diagnosis and vascular patterns

All 113 patients were diagnosed with n-SCLC. After staining for the endothelial marker CD31, nine cases were classified as putative non-angiogenic and 104 as angiogenic. Non-angiogenic cases accounted for 8.6% of the total cases. This frequency is consistent with the frequencies described so far in two other series: 16% (Pezzella *et al*, 1997) and 12% (Offersen *et al*, 2001).

### Evaluation of the vascular phenotype

#### Normal lung

**Figure 1:**
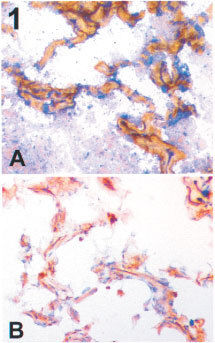
Normal lung (frozen sections). (**A**) (×400) Double immunostaining for LH39 (in brown) and CD31 (in blue); no counterstaining was done. The vessels of the alveolar walls have LH39 expression in their membrane. (**B**) (×400) Staining for αVβ3 (in brown). The nuclei are counter stained with haematoxilin (blue). A variable positivity on the endothelial cells is present.

Normal lung from neoplastic and non-neoplastic patients showed positive staining of the basal membrane for LH39. The staining of endothelial cells with anti αVβ3 antibodies was variable with the endothelial cells in some cases being negative but in others weakly or even strongly positive (Figure 1

).

#### Non-angiogenic tumours

**Figure 2:**
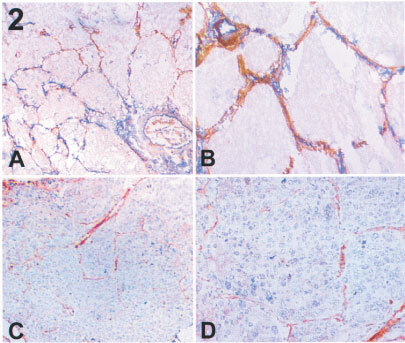
A non-angiogenic tumour (frozen sections). (**A**) (×100) (low magnification) and (**B**) (×400) double immunostaining for LH39 (in brown) and CD31 (in blue); no counterstaining was done. All the vessels express LH39 in the basal membrane. (**C**) (×100), (**D**) (×400) Immunostaining for αVβ3 (in brown); the nuclei are counter stained with haematoxilin (blue). A variable positivity on the endothelial cells is present as seen in normal lung (Figure 1).

In the nine putative non-angiogenic cases investigated, all the intratumoral vessels showed a basal membrane positive for LH39. The endothelial cells were negative, weakly stained or even focally strongly positive, with anti αVβ3 antibody, in a fashion similar to that observed in normal lung (Figure 2

).

#### Angiogenic tumours

*Heterogeneity of newly formed vessels:* In all, 104 cases of angiogenic carcinomas were studied. In 102 cases double immunostaining for CD31 and LH39 was obtained while a readable immunostaining for αVβ3 was obtained in only 44 cases. In total, staining for all antibodies was assessable in 42 angiogenic tumours.

If the hypothesis is true that the growth of newly formed vessels (LH39 negative) is dependent on the expression of αVβ3, then the two percentages should complement each other in that their case-wise sum should be 100. In other words all the LH39 negative vessels would be αVβ3 positive.

**Figure 3:**
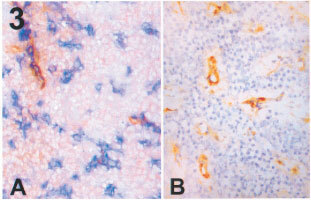
An angiogenic lung carcinoma (frozen sections). (**A**) (×400) double immunostaining for LH39 (in brown) and CD31 (in blue) no counter staining was done. Only a few vessels express LH39 in the basal membrane. (**B**) (×400) Immunostaining for αVβ3 (in brown). The nuclei are counter stained with haematoxilin (blue). Some vessels are strongly stained.

The immunostaining patterns of a typical angiogenic tumour are seen in Figure 3

**Figure 4:**
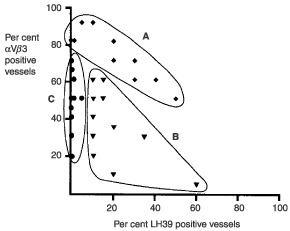
Phenotype of intratumour vessels in 44 angiogenic lung n-SCLC. Three groups of tumours can be identified. Groups A (diamonds) and B (triangles) are made up of a mixture of mature (LH39 positive) and immature (LH39 negative) vessels. However while in group A all the immature express αVβ3, in group B the immature are, in part, αVβ3 negative. Group C (circles) is made up almost completely by new vessels (LH39 negative) but only a proportion are αVβ3 positive. N.B.: each symbol in the diagram may well represent more than one sample.

. The average percentage of LH39 positive (mature) intra-tumoural vessels is 12.9% (range 0–60%) while the average percentage of αVβ3 positive (newly formed) vessels is 54% (range 5–90%). In Figure 4

the percentages of mature (LH39 positive) *vs* immature (defined as αVβ3 positive) are plotted. An inverse correlation is expected if the assumption that all the newly formed vessels are αVβ3 positive is true. It is obvious that the hypothesis does not hold for the whole of the data set as, in some cases, some vessels are immature (LH39 negative) but do not express αVβ3.

Closer observation (Figure 4) reveals that of 42 angiogenic carcinomas only 14 (group A) fulfil the theoretical criteria of a sum of 100% (that is about one third of the data set) whereas the majority of the cases have a sum of 81% or less (groups B and C). In group B (15 cases) the sum of vessels positive for LH39 and/or αVβ3 was between 5 and 75%. In the remaining 13 cases (group C) no LH39 staining was detected in the basal membrane of the vessels, but still only a proportion was αVβ3.

*The angiogenic switch:* This study of vessel phenotype highlights that in 17 out of the cases investigated, a residual component of pre-existent vessels is seen in addition to neo-angiogenesis.

**Figure 5:**
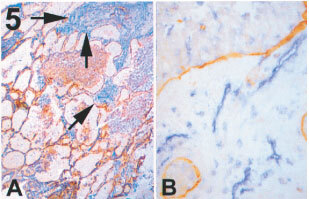
Angiogenic tumour, basal pattern with residual non angiogenic component (frozen sections). Double immunostaining for LH39 (in brown) and CD31 (in blue), no counter staining was done. (**A**) (×100) In some areas an alveolar pattern is still present. The arrows indicates where tumour-associated stroma is present and where remodelling and angiogenesis start. (**B**) (×400) Islands of neoplastic cells survive surrounded by stroma with new vessels.

In 14 cases out of these 17 tumours, the tumour had a predominant angiogenic pattern classified as basal according to our described criteria (Pezzella *et al*, 1997), however the outer area of the neoplastic nodule shows that the neoplastic cells are exploiting the pre-existing alveolar vessels (CD31 and LH39 positive). Deeper in the tumour the alveolar walls appear to become oedematous, infiltrated by lymphocytes and macrophages. Among them numerous immature microvessels, negative for LH39, are present (Figure 5

).

**Figure 6:**
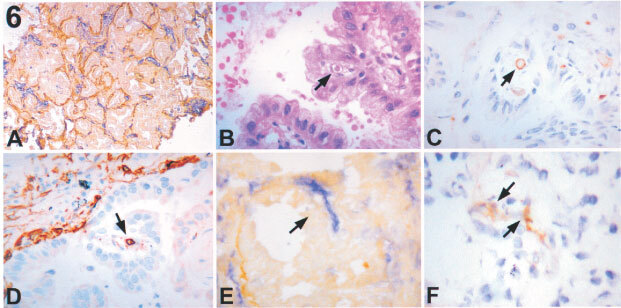
Early papillary pattern (frozen sections). (**A**) (×40) Double immunostaining for LH39 (in brown) and CD31 (in blue). No counter staining was done. The alveolar pattern appears preserved throughout the tumour. (**B**) (×100) Haematoxilin and Eosin (**C**) (×100) CD31 (in brown, the nuclei are counter stained with haematoxilin (blue)) and (**D**) (×100) CD34 (in brown, the nuclei are counter stained with haematoxilin (blue)). Within these micropapillae a small vessel (arrow), positive for CD31 and CD34, is present. (**E**) (×100) Double immunostaining for LH39 (in brown) and CD31 (in blue). No counter staining was done. A new vessel CD31 positive but LH39 negative (arrow) is present in the stalk of this micropapilla. (**F**) (×100) Staining for αVβ3 (in brown). The nuclei are counter stained with haematoxilin (blue). An αVβ3 positive microvessel (arrow) within an early papilla.

A different pattern of angiogenic switch is instead observed in the remaining three cases of tumours. These cases had a papillary pattern (Pezzella *et al*, 1997). However the papillary component accounts for the minority of the tumour so we have termed it ‘early papillary’. As shown in Figure 6

, the columnar cells of these three cases grow along CD31 and LH39 positive alveolar vessels. However, within some alveolar spaces, small buds of neoplastic cells occur with a central fibrous core in which LH39 negative αVβ3 positive vessels are present.

## DISCUSSION

The relationship between tumour and vessels is a complex one. As it happens frequently, diverse experimental evidence has been produced supporting apparently contradictory hypotheses, which may all be partly true but in a wider and more complex context than initially thought. An early hypothesis stated that tumours were associated with pre-existing vessels (Coman and Sheldon, 1946; Warren, 1979). Later robust experimental evidence (for review see Folkman, 1995) was produced illustrating the fundamental role of new vessel formation in neoplastic growth. However, the model that neo-angiogenesis is necessary for a tumour to become larger than a few millimetres, and hence become clinically detectable, does not appear to hold for all tumours.

The present study has produced two main results: the first concerns the phenotype of the intra-tumoural vessels in the non-angiogenic tumours, the second the phenotype of the vessels in the angiogenic tumours. As far as the first point is concerned we wanted to establish whether in some primary lung tumours in which there is no morphological evidence of neo-angiogenesis, the vessels have a phenotype comparable to that of the vessels present in normal lung or to that of newly formed vessels. Having characterized the intra-tumoural vessels using the LH39 and αVβ3 markers, we found a clear result: the vessels present in the putative non-angiogenic lung carcinomas not only have the same architectural pattern, but also the same phenotype, as those present in normal lung. We could not find vessels with an immature phenotype in these tumours.

While our data confirm the novel finding that some tumours can grow to clinically detectable dimensions in the absence of angiogenesis, they also confirmed some observations previously reported in the literature regarding the vascular phenotype of normal vessels. We confirmed (Almeida *et al*, 1992a) that vessels of the normal lung express LH39 in their basal membrane. In contrast staining for αVβ3 was not an absolute marker of newly formed vessels. While it is well established that endothelial expression of αVβ3 is essential for angiogenesis induced by basic fibroblastic growth factor or tumour necrosis factor α (Brooks *et al*, 1994; Friedlander *et al*, 1995), it has also been found that it can be up-regulated in resting endothelium (Tang *et al*, 1994). Studies in normal human tissue have shown that endothelium is negative for αVβ3 in breast (Gasparini *et al*, 1998) while in normal brain, skin and kidney the endothelial cells express it to various degrees (Rabb *et al*, 1996; Navratil *et al*, 1997; Pazouki *et al*, 1997). It is arguable whether this is due to the nearby tumour: we observed the same pattern in normal lung from neoplastic and non-neoplastic patients.

Our second set of results is concerned with the vascular phenotype in angiogenic tumours. In all the cases classified as angiogenic we have confirmed that most of the vessels have an immature phenotype, with deficient LH39 expression, as observed previously in oral carcinoma (Almeida *et al*, 1992b), breast carcinoma (Kakolyris *et al*, 2000), and another series of n-SCLC (Kakolyris *et al*, 1999). It should be noted that a variable number of mature vessels is still present.

Furthermore, we showed that in a number of cases, the newly formed vessels are likely to be driven by at least two pathways: one being dependent on αVβ3 and the other being independent. Such a heterogeneity raises a question concerning the response to anti-angiogenic treatment: within the same tumours some vessels would respond to such treatments (e.g. the humanized anti αVβ3 antibody Vitaxin (Gutheil *et al*, 2000) whilst others would not.

A final point concerns the topographical localization of where in the tumour the angiogenic switch could occur. This is now made possible by the ability to distinguish recently formed vessels from pre-existent ones. Whilst discussing the angiogenic switch Hanahan and Folkman (1996), raised the following questions:

- When is angiogenesis activated during the development of a tumour?
- Is angiogenesis simply an inevitable consequence of any nodules of aberrant proliferating cells becoming size-limited by lack of vascularization or is it a discrete component of the tumour phenotype?

As far as the first question is concerned, Hanahan and Folkman (1996) showed convincing evidence that both in experimental murine and in human tumours there was a dramatic difference in microvessel density between early hyperplasia and neoplastic transformation. These observations indicated that the angiogenic switch occurs at an early stage of tumour growth. Studies of human tumours (breast, cervix and lung) indicate that the occurrence of high microvessel density is detected in association with carcinoma *in situ* and precedes the occurrence of infiltration. In contrast the patterns of vessel distribution that we observed suggest that in some circumstances angiogenic switching can occur at a later stage, further demonstrating that in these tumours the angiogenic switch is not necessary for the tumour itself to grow. This is not surprising in view of our other finding that some tumours can grow without any angiogenesis at all. Furthermore, the delayed occurrence of the angiogenic switch has been shown to occur in a rat model by Holash *et al* (1999a). These observations raise the question of why in these tumours angiogenesis is occurring at all.

This point brings us to the second question raised by Hanahan and Folkman (1996): is the angiogenic switch just a step necessary for the neoplastic growth or is it a discrete component in the tumour phenotype? Our finding that, both in some squamous carcinomas and in adenocarcinomas of the lung, the switch can occur when the tumour is already expanding, further supports the conclusion of Hanahan and Folkman (1996) that the angiogenic switch is a discrete component of the tumour phenotype.

Whether primary lung non-angiogenic tumours are more aggressive than angiogenic tumours is debated (Pastorino *et al*, 1997; Offersen *et al*, 2001) but they appear to be anyway fairly aggressive tumours and vessels could still play a key role. The normal vessels trapped in the tumour could be more effective than the newly formed for several reasons. They could allow the tumour to growth efficiently by exploiting the highly regular vascular network of the lung and progressing by filling the empty alveolar spaces. Lung vessels also offer a ‘vascular window’ which can probably favour neoplastic spread.

We have also described lung metastases with a non-angiogenic tumour appearing after a long disease-free interval (Pezzella *et al*, 2000). This implies that neoplastic cells could escape dormancy even if angiogenesis is still suppressed.

Finally the finding that primary and secondary tumours can be non-angiogenic raises the issue of potential resistance to anti-angiogenic treatments and highlights the need for tailor-made treatments against such tumours.

## References

[bib1] AlmeidaBMChallacombeSJEvesonJWSmithCGLeighIM1992a A novel lamina lucida component of epithelial and endothelial basement membranes detected by LH39 monoclonal antibody J Pathol 166 243253 1517880 10.1002/path.1711660306

[bib2] AlmeidaBMChallacombeSJEvesonJWMorganPRPurkisPELeighIM1992b The distribution of LH39 basement membrane epitope in the tumour stroma of oral squamous cell carcinomas J Pathol 166 369374 1381430 10.1002/path.1711660408

[bib3] BrooksPCClarkRAFChereshDA1994 Requirement of vascular integrin aVb3 for angiogenesis Science 264 569571 7512751 10.1126/science.7512751

[bib4] ComanDRSheldonWF1946 The significance of hyperemia around tumour implants Am J Pathol 22 821 20991978

[bib5] FolkmanJ1971 Tumour angiogenesis: therapeutic implication N Engl J Med 285 11821186 4938153 10.1056/NEJM197111182852108

[bib6] FolkmanJ1990 What is the evidence that tumours are angiogenesis dependent JNCI 82 46 1688381 10.1093/jnci/82.1.4

[bib7] FolkmanJ1995 Tumour angiogenesisInThe molecular basis of cancerMendelshon J, Howley PM, Israel MA, Liotta LA (eds).pp206232Philadelphia, PA: W.B. Saunders

[bib8] FriedlanderMBrooksPCShafferRWKincaidCMVarnerJACheresDA1995 Definition of two angiogenic pathways by distinct alpha v integrins Science 270 15001502 7491498 10.1126/science.270.5241.1500

[bib9] GaspariniGBrooksPCBiganzoliEVermeulenPBBonoldiEDirixLYRanieriGMiceliRChereshDA1998 Vascular integrin avb3: a new prognostic indicator in breast cancer Clin Cancer Res 4 26522634

[bib10] GutheilJCCampbellTNPiercePRWatkinsJDHuseWDBodkinDJChereshDA2000 Targeted antiangiogenic therapy for cancer using vitaxin: A humanized monoclonal antibody to the integrin alpha(nu)beta(3) Clin Cancer Res 6 30563061 10955784

[bib11] HanahanDFolkmanJ1996 Patterns and emerging mechanisms of the angiogenic switch during tumorigenesis Cell 86 353364 8756718 10.1016/s0092-8674(00)80108-7

[bib12] HolashJMaisonpierrePCComptonDBolandPAlexanderCRZagzagDYancopoulosGDWiegandSJ1999a Vessel cooption, regression, and growth in tumours mediated by angiopoietin and VEGF Science 284 19941998 10373119 10.1126/science.284.5422.1994

[bib13] HolashJWiegandSJYancopoulosGD1999b New model of tumor angiogenesis: dynamic balance between vessel regression and growth mediated by angiopoietins and VEGF Oncogene 18 53565362 10498889 10.1038/sj.onc.1203035

[bib14] HyjekEChadburnADiasSZhuZWitteLHicklinDCesermanEKnowelsDRafiiS1999 High grade Non-Hodgkin's lymphomas and Hodgkin's disease are associated with increase density of KDR+SMA(-) immature microvessels Blood 94Suppl 12659

[bib15] KakolyrisSGiatromanalakiKoukourakisMLeighIMGeorgouliasVKanavarosPSivridisEGatterKCHarrisAL1999 Assesment of vascular maturation in non small cell using a novel basement membrane component, LH39: correlation with p53 and angiogenic factor expression Cancer Res 59 56025607 10554041

[bib16] KakolyrisSFoxSBKoukourakisMGiatromanolakiABrownNLeekRDTaylorMLeighIMGatterKCHarrisAL2000 Relationship of vascular maturation in breast cancer blood vessels to vascular density and metastasis, assessed by expression of a novel basement membrane component, LH39 Br J Cancer 82 844851 10732757 10.1054/bjoc.1999.1010 PMC2374391

[bib17] KolinAKoutoulakisT1988 Role of arterial occlusion in pulmonary scar cancers Hum Pathol 19 11611167 3169724 10.1016/s0046-8177(88)80147-3

[bib18] KolinA1995 Tumor angiogenesis in human lung adenocarcinoma Cancer 76 151 10.1002/1097-0142(19950701)76:1<151::aid-cncr2820760123>3.0.co;2-d 8630867

[bib19] ManiotisAJFolbergRHessASeftorEAGardnerLMGPe'erJTrentJMMeltzerPSHendrixMJC1999 Vascular channel formation by human melanoma cells in Vivo and in Vitro: vasculogenic mimicry Am J Pathol 155 739752 10487832 10.1016/S0002-9440(10)65173-5 PMC1866899

[bib20] NaganoNSasakiHAoyagiMHirakawaK1993 Invasion of experimental rat brain tumor: early morphological changes following microinjection of C6 glioma cells Acta Neuropathol Berl 86 117125 8213067 10.1007/BF00334878

[bib21] NavratilECouvelardAReyAHeninDScoazecJY1997 Expression of cell adhesion molecules by microvascular endothelial cells in the cortical and subcortical regions of normal human brain: an immunohistochemical analysis Neuropathol Appl Neurobiol 23 6880 9061692

[bib22] OffersenBVPfeifferPHamilton-DutoitSOvergaardJ2001 Patterns of angiogenesis in nonsmall-cell hung carcinoma Cancer 91 15001509 11301398

[bib23] ParumsDVCordellJLMicklemKHeryetARGatterKCMasonDY1990 JC70: a new monoclonal antibody that detects vascular endothelium associated antigen on routinely processed tissue sections J Clin Pathol 43 752757 2212067 10.1136/jcp.43.9.752 PMC502755

[bib24] PastorinoUAndreolaSTagliabueEPezzellaFIncarboneMSozziGBuyseMMenardSPierottiMRilkeF1997 Immunocytochemical markers in stage I lung cancer (NSCLC): relevance to prognosis J Clin Oncol 15 28582865 9256129 10.1200/JCO.1997.15.8.2858

[bib25] PazoukiSChisolmDMAdiMMCarmichaelGFarquharsonMOgdenGRSchorSLSchorAM1997 The association between tumour progression and vascularity in the oral mucosa J Pathol 183 3943 9370945 10.1002/(SICI)1096-9896(199709)183:1<39::AID-PATH1088>3.0.CO;2-L

[bib26] PezzellaFDi BaccoAAndreolaSNicholsonAGPastorinoUHarrisAL1996 Angiogenesis in primary lung cancer and lung secondaries Eur J Cancer 32A 24942500 9059338 10.1016/s0959-8049(96)00377-2

[bib27] PezzellaFPastorinoUTagliabueEAndreolaSSozziGGaspariniGMenardSGatterKCHarrisALFoxSBuyseMPilottiSPierottiMRilke F1997 Non-small cell lung carcinoma tumour growth without neo angiogenesis Am J Path 5 14171423 PMC1858069 9358768

[bib28] PezzellaFManzottiMDi BaccoAVialeGNicholsonAGPriceRRatcliffeCPastorinoUGatterKCAltmanDGHarrisALPilottiSVeronesiU2000 Evidence for a novel non-angiogenic pathway in breast cancer metastasis Lancet 355 17871788 10832831

[bib29] RabbHBarroso-VicensEAdamsRPow-SangJRamirezG1996 Alpha-V/beta-3 and alpha-V/beta-5 integrin distribution in neoplastic kidney Am J Nephrol 16 402408 8886177 10.1159/000169032

[bib30] TangDGDiglioCAHonnKV1994 Activation of microvascular epithelium by ecosanoid 12(S)-hydroxyeicosatetraeonic acid leads to enhanced tumor cell adhesion via up-regulation of surface expression of alpha v beta 3 integrin: a postranscriptional, protein kinase C- and cytoskeleton-dependent process Cancer Res 54 11191129 8313370

[bib31] WarrenBA1979 The vascular morphology of tumorsInTumor blood circulation: Angiogenesis, vascular morphology and blood flow of experimental human tumorsPeterson H-I (ed)pp147FL: CRC Press

[bib32] WesselingPvan der LaakJAde LeeuwHRuiterDJBurgerPC1994 Quantitative immunohistological analysis of the microvasculature in untreated human glioblastoma multiforme. Computer-assisted image analysis of whole tumor sections J Neurosurg 81 902909 7525899 10.3171/jns.1994.81.6.0902

